# Association of kidney function with physical performance: the Maastricht study

**DOI:** 10.1007/s40620-024-01933-8

**Published:** 2024-04-09

**Authors:** Ioannis Bellos, Smaragdi Marinaki, Pagona Lagiou, Ioannis N. Boletis, Annemarie Koster, Marleen M. J. van Greevenbroek, Simone J. P. M. Eussen, Hans H. C. M. Savelberg, Anke Wesselius, Vassiliki Benetou

**Affiliations:** 1 Department of Hygiene, Epidemiology and Medical Statistics, School of Medicine, National and Kapodistrian University of Athens, Athens, Greece; 2 Department of Nephrology and Renal Transplantation, Laiko General Hospital, National and Kapodistrian University of Athens, 75, Mikras Asias Str., 11527 Athens, Greece; 3 CAPRHI Care and Public Health Research Institute, Maastricht University, Maastricht, The Netherlands; 4 Department of Social Medicine, Maastricht University, Maastricht, The Netherlands; 5 Department of Human Biology and Movement Science, NUTRIM School for Nutrition and Translational Research in Metabolism, Maastricht University Medical Centre+, PO Box 616, 6200 MD Maastricht, The Netherlands; 6 Department of Epidemiology, Maastricht University, Maastricht, 6229ER The Netherlands; 7 CARIM School for Cardiovascular Diseases, Maastricht University, Maastricht, 6229ER The Netherlands; 8 School of Nutrition and Translational Research in Metabolism, Maastricht University, Maastricht, 6229ER The Netherlands

**Keywords:** Glomerular filtration rate, Albuminuria, Physical performance, Muscle strength

## Abstract

**Background:**

Kidney failure has been associated with decreased physical capacity, although evidence regarding the physical performance of individuals with earlier stages of chronic kidney disease (CKD) remains limited.

**Methods:**

Cross-sectional data were derived from the prospective, population-based Maastricht Study. Multivariate linear regression models were fitted to assess the association of estimated glomerular filtration rate (eGFR) and albuminuria categories with physical performance test outcomes.

**Results:**

Overall, 7396 participants were included. Compared to eGFR 60–90 ml/min/1.73 m^2^, values < 60 ml/min/1.73 m^2^ were associated with significantly shorter 6-min walk distance (*β*: − 13.04 m, 95% confidence intervals-CI − 19.95; − 6.13), worse timed chair rise stand test time (*β*: 0.91 s, 95% CI 0.36; 1.47), lower maximal grip (*β*: − 0.83 kg, 95% CI − 1.50; − 0.15) and elbow flexion (*β*: − 3.64 Nm, 95% CI − 7.11; − 0.16) strength. Additionally, eGFR > 90 ml/min/1.73 m^2^ was linked to significantly shorter 6-min walk distance (*β*: − 6.13 m, 95% CI − 9.44; − 2.82). Urinary albumin excretion > 30 mg/24 h was associated with shorter 6-min walk distance (*β*: − 12.48 m, 95% CI − 18.28; − 6.68), worse timed chair rise stand test time (*β*: 0.51 s, 95% CI 0.11; 1.06), lower maximal grip (*β*: − 1.34 kg, 95% CI − 1.91; − 0.76) and elbow flexion strength (*β*: − 3.31 Nm, 95% CI − 5.80; − 0.82).

**Conclusions:**

Reduced eGFR and higher albuminuria levels were associated with worse physical performance, especially shorter 6-min walk distance and lower muscle strength. The relationship between eGFR and physical function was non-linear, with also high eGFR values being associated with worse performance, especially in the six-minute walk test.

**Graphical abstract:**

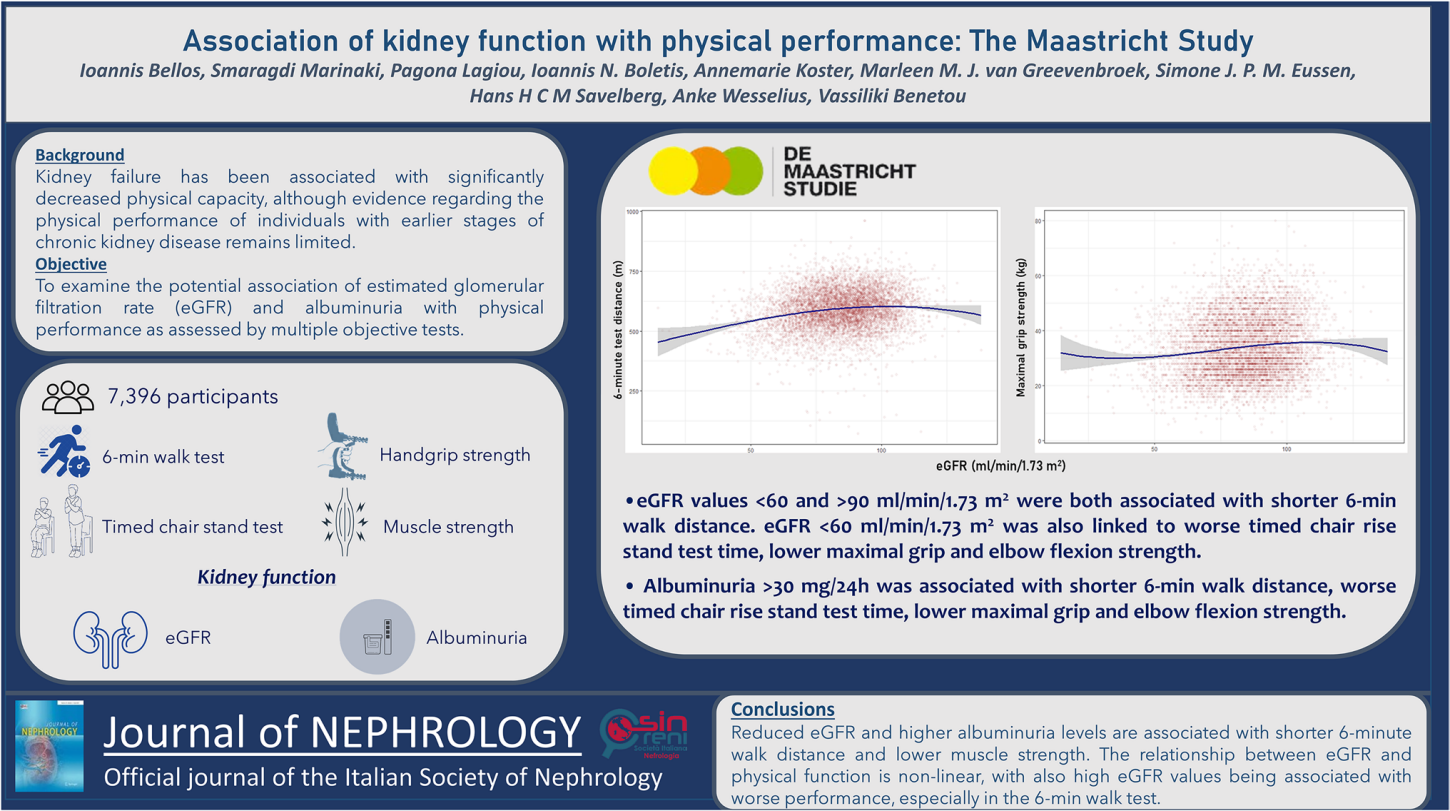

**Supplementary Information:**

The online version contains supplementary material available at 10.1007/s40620-024-01933-8.

## Introduction

Impaired physical function represents a rising public health concern in the aging population, as it is associated with increased rates of morbidity and mortality [1]. Loss of muscle strength and the development of sarcopenia have been linked to high health costs, worse quality of life and poor mental health, contributing to accelerated cognitive decline [2, 3]. The pathophysiology of reduced physical performance is complex, as it is based on the interplay of genetic, hormonal, inflammatory and environmental factors, such as diet and energy intake [4]. Measures of physical function have been suggested to have a significant prognostic role in various clinical entities, such as heart failure [5], chronic obstructive pulmonary disease [6] and diabetes mellitus [7].

Physical performance can be assessed subjectively via questionnaires [8] and objectively with the use of tests evaluating various parameters, such as gait speed, grip and isometric muscle strength. Specifically, gait speed reflects the strength of lower extremities in conjunction with the status of vision and cognitive function, representing an inexpensive marker able to predict shorter survival and cardiovascular disease risk among older adults [9]. On the other hand, the six-minute walk test requires no specific equipment and is appropriate even for individuals with already acquired poor functional status, such as patients with chronic obstructive pulmonary disease and heart failure [10]. In addition, grip strength has been proposed as a measure of overall strength, correlating with the risk of all-cause and cardiovascular mortality [11]. The timed chair rise stand test evaluates a combination of speed, lower body strength and balance and provides information about both the musculoskeletal and somatosensory systems, reflecting the degree of frailty and predicting the risk of falls in older adults [12, 13].

End-stage kidney disease necessitating renal replacement therapy has been associated with significantly decreased physical capacity, due to a combination of multiple factors, such as increased protein catabolism, mineral bone disease and malnutrition [14]. However, current evidence regarding the physical performance of individuals with earlier stages of chronic kidney disease (CKD) remains limited, providing contradictory outcomes regarding the association between kidney function and physical performance. The present study aims to shed more light on the potential link between kidney function and physical performance by evaluating the association of glomerular filtration rate and urinary albumin excretion with objective markers of physical performance.

## Materials and methods

### Study population and design

This is a cross-sectional analysis of data derived from the Maastricht study population. The Maastricht Study is a prospective, population-based, cohort study performed in the southern part of the Netherlands. The methodology of the study has been previously described [15]. In brief, the Maastricht Study follows an extensive phenotyping approach, aiming to examine the pathophysiology and complications of type 2 diabetes mellitus. Recruitment of individuals aged between 40 and 75 years was performed with stratification based on diabetes mellitus status, with oversampling of those with known type 2 diabetes mellitus. Participants were recruited using mass media campaigns, municipal registries and the regional Diabetes Patient Registry via mailings. This analysis is based on participants who completed the baseline survey (November 2010 to September 2013) providing available data regarding both kidney and physical function. All individuals were fully informed about the study procedures and provided written informed consent. The study procedures were ethically approved by the institutional medical ethical committee (NL31329.068.10) and the Netherlands Health Council (Permit 131,088–105234-PG).

### Physical performance

Participants’ physical performance was quantified using the following tests: 6-min walk test, timed chair rise stand test, hand grip strength and isometric strength tests of elbow and knee flexors and extensors.

For the 6-min walk test, participants were instructed to walk at a fast pace without running for 6 min, making turns around two cones, placed 20 m apart in a hallway. Every minute, participants were encouraged to continue walking. After the end of 6 min or when the individual was unable or unwilling to continue, the test was terminated and the covered distance was measured (in meters). For the analyses, the covered distance (m) and the gait speed (s) were captured. For the six-minute walk test the following exclusion criteria applied: history of cardiovascular disease 3 months prior to the test, serious hypertension (systolic blood pressure ≥ 180 mmHg and/or diastolic blood pressure ≥ 110 mmHg), tachycardia (heart rate ≥ 110/min) and mobility limitation, such as the need of a walker or any other condition precluding independent walking.

For the timed chair rise stand test, a 46 cm-high chair with a straight back without armrests was used. Participants were instructed to sit in the middle of the chair, keeping their feet flat on the floor and their arms crossed over the chest. The test required participants to come to a full standing position and sit down again without the support of their arms at the fastest possible pace. For the analysis, the time in seconds needed for the completion of 10 repetitions was measured to one decimal place [16, 17].

Regarding the measurement of handgrip strength, the Jamar handheld dynamometer (SEHAN Corp., Korea-Biometrics Europe BV, Almere) was used. Participants were required to stand straight against a wall, having their upper arm along the trunk with their elbow flexed at 90°. Subsequently, they were instructed to strongly squeeze the dynamometer for 3–5 s, while standardized encouragement was provided. Three measurements were performed for each hand and the maximum strength measurement was recorded in kilograms.

Isometric muscle strength of knee and elbow extensors and flexors was evaluated using a customized set-up with two dynamometers (Futek LSB302, FUTEK Advanced Sensor Technology Inc., Irvine, CA, USA) and was recorded with the M-PAQ (Maastricht Instruments, Maastricht, the Netherlands). Individuals were excluded in case of recent injury or surgery of the right arm or leg. Straps connected to the dynamometer were secured 2 cm above the lateral malleolus and 2 cm proximally from the wrist, with the axis of the dynamometer corresponding to the knee-joint and the elbow-joint axis, respectively. Participants were instructed to flex and extend their right knee and elbow powerfully for 5 s. Three measurements were performed, while the generated force was visualized on a monitor. For the analysis, the joint torques were calculated by multiplying the force measured by the dynamometer by the corresponding moment-arm, defined as the distance between the dynamometer strap and the joint rotation point. The maximal joint torques out of the three trials for elbow and knee flexion and extension were captured in Nm.

### Kidney function

The estimated glomerular filtration rate (eGFR) was calculated with the Chronic Kidney Disease Epidemiology Collaboration (CKD-EPI) equation, which is based on the combination of serum creatinine and serum cystatin-C values (eGFR_Cr-Cys_) [18]. In case serum cystatin-C measurements were not available, the creatinine-based CKD-EPI equation was used (eGFR_Cr_). For the analyses, eGFR was classified as follows: < 60, 60 to 90 and > 90 ml/min/1.73 m^2^. Renal hyperfiltration was defined as an eGFR exceeding the age-adjusted threshold that was calculated as follows: 130 ml/min/1.73 m^2^ minus 1 ml/min/1.73 m^2^ per year after the age of 40 years [19]. Urinary albumin excretion was measured by two 24-h urine collections. Valid collections were considered those with collection times between 20 and 28 h, with extrapolation to the 24-h excretion performed when the collection time was not exactly 24 h. Urinary albumin excretion was categorized as < 15 mg/24 h, 15–30 mg/24 h and > 30 mg/24 h. According to the KDIGO (Kidney Disease: Improving Global Outcomes) definitions, eGFR values < 60 ml/min/1.73 m^2^ indicated a moderate to severe kidney function decrease (stage ≥ G3a), while urinary albumin excretion > 30 mg/24 h implied moderately to severely increased albuminuria (stage ≥ A2). On the other hand, eGFR > 90 ml/min/1.73 m^2^ indicated normal to high eGFR[20].

### Covariates

Self-reported information was recorded with the use of questionnaires regarding age, sex, ethnicity (Caucasian/other), educational level, smoking habits and total alcohol consumption (in g per day). More specifically, low education referred to primary or lower vocational education. Medium education was defined as intermediate vocational or higher secondary education, while the high level of education corresponded to higher vocational or university education. High alcohol consumption was defined as the intake of more than 7 g and 14 g of alcohol per day for females and males, respectively. The status of diabetes mellitus was assessed by a 7-point 75 gr oral glucose tolerance test. Glucose metabolism was categorized as normal, prediabetes, or type 2 diabetes according to the 2006 World Health Organization criteria [21]. Individuals on anti-diabetic medications without a history of type 1 diabetes were classified as having type 2 diabetes. Daily energy intake (in kcal/day) was estimated by a validated food frequency questionnaire [22], body mass index was estimated by dividing weight (in kilograms) by the square of height (in meters) and waist-to-hip ratio by dividing waist by hip circumference (in cm). Information about mobility status was captured by the 36-Item Short Form Health Survey questionnaire [8], defining mobility limitation as the difficulty in climbing one flight of stairs or walking 500 m. Fasting blood samples were collected to assess the lipid profile of participants. To evaluate office blood pressure, three measurements were performed on the right arm after a 10-min rest and the average systolic and diastolic blood pressure values were recorded. Hypertension was defined by one of the following criteria: average systolic blood pressure ≥ 140 mmHg, average diastolic blood pressure ≥ 90 mmHg [23], or use of anti-hypertensive medications.

### Statistical analysis

Statistical analysis was performed in R-4.0.5. Histograms were visually assessed to examine the distribution of continuous variables. Normally distributed variables were described by their mean and standard deviation; otherwise, the median and interquartile range (IQR) were reported. Comparisons of continuous variables across 3 groups were planned to be conducted using the one-way analysis of variance or the non-parametric Kruskal Wallis test, depending on data normality. Categorical variables were reported as proportions and were statistically compared using the chi-square test or the Fisher’s exact test in case the assumptions of the former were not fulfilled.

Scatterplots were constructed to assess the linearity of association between kidney and physical function markers. To assess non-linear relationships, restricted cubic splines regression analysis was performed with 4 knots placed at the 5th, 35th, 65th and 95th percentiles. In linear regression models, eGFR and urinary albumin excretion were treated as categorical variables, while physical function markers as continuous ones. Participants with eGFR 60–90 ml/min/1.73 m^2^ and urinary albumin excretion < 15 mg/24 h served as the reference groups. Multivariable linear regression models were constructed aiming to take into consideration potentially important covariates, such as basic demographic data, variables that could possibly impact physical function, as well as diabetes mellitus status to account for oversampling. Adjustment for mobility limitation was performed to account for physical disability that would interfere with the proper evaluation of physical performance tests. Specifically, model 1 adjusted sequentially for age, sex, ethnicity (Caucasian or other), educational level (low, medium or high) and diabetes mellitus status (normal glucose metabolism, prediabetes, type 2 diabetes mellitus or other diabetes), model 2 adjusted additionally for body mass index, smoking (never, former or current smoker), mobility limitation and alcohol consumption and model 3 additionally adjusted for systolic blood pressure. Albuminuria models also adjusted for eGFR. Moreover, as an additional analysis, the potential association of physical performance markers with renal hyperfiltration (age-adjusted eGFR > 130 ml/min/1.73 m^2^) was tested. Participants with renal hyperfiltration were compared to normofiltering ones (age-adjusted eGFR: 60–90 ml/min/1.73 m^2^). Subgroup analysis was performed by separately analyzing the association of physical function markers with eGFR_Cr-Cys_ and eGFR_Cr_. In addition, stratified analyses were performed based on sex and diabetes mellitus status, testing for the presence of potential interactions. Sensitivity analysis was also conducted by repeating the regression analysis in patients without hypertension and those without dyslipidemia requiring lipid-modifying medications. Statistical significance was defined by a two-sided *p-value* threshold of 0.05.

## Results

### Study population

**Fig. 1:**
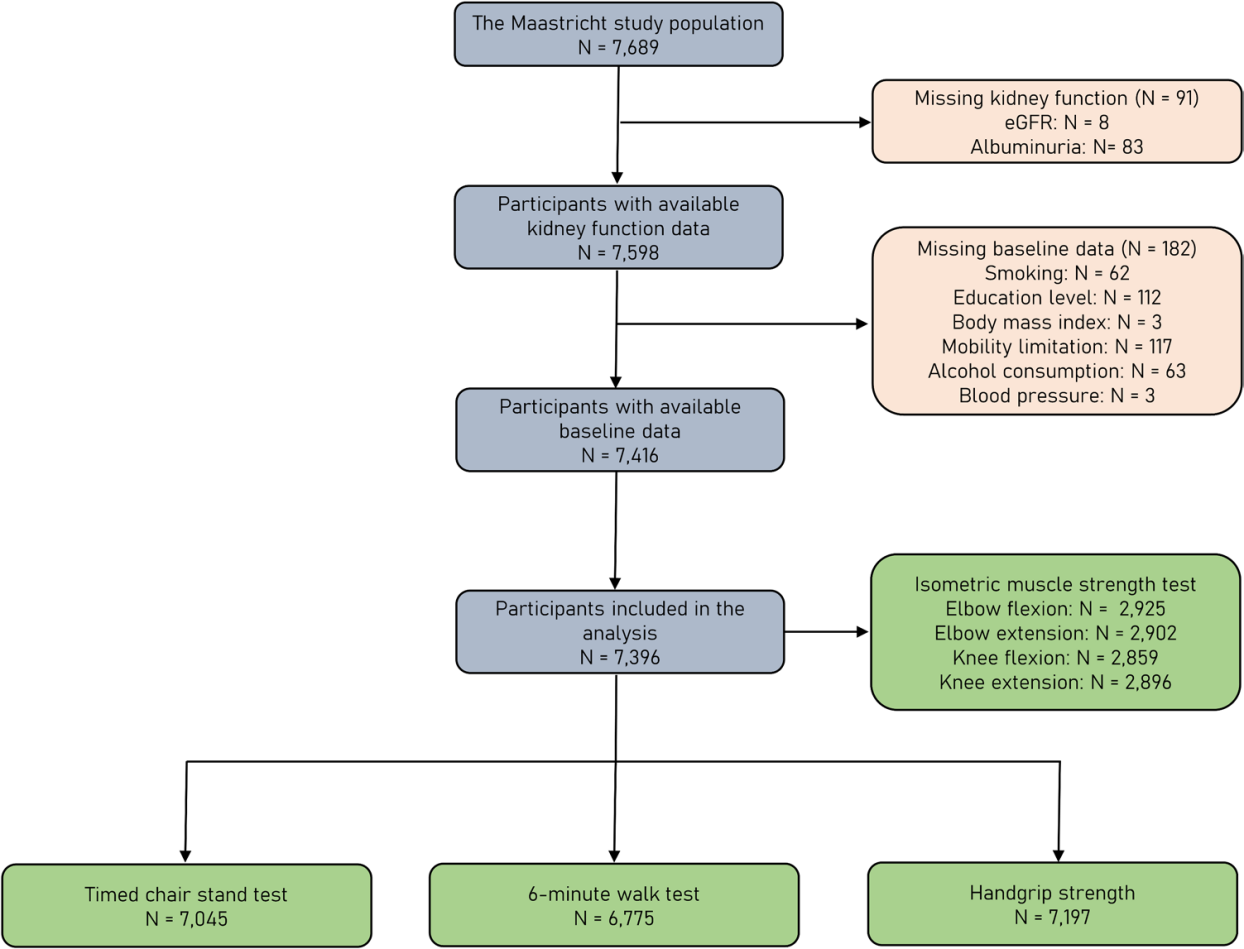
Flowchart of participant inclusion

The selection of participants is schematically illustrated in Fig. 1. Overall, 296 individuals were completely excluded from the analysis due to missing data about kidney function (*n* = 91), baseline covariates (*n* = 182) and physical function tests (*n* = 20); hence, a total of 7,396 participants were included in the present analysis. The clinical and demographic characteristics of included and excluded individuals are compared in Suppl. Table 1. Kidney function was assessed by the CKD-EPI eGFR_Cr-Cys_ equation in 3,249 participants with available cystatin-c measurements.

Table 1 presents the main demographic, clinical and physical function characteristics of the included participants, stratified by eGFR categories. The median age of the study population was 61 years, while 50.5% of participants were males. Concerning kidney function, 5.8% of participants had an eGFR < 60 ml/min/1.73 m^2^ and 8.4% of participants had a urinary albumin excretion > 30 mg/24 h. The median eGFR was 84.70 ml/min/1.73 m^2^, while hyperfiltration was observed in 86 participants. More specifically, eGFR 45–60 ml/min/1.73 m^2^ was present in 62 individuals, while 8 participants had an eGFR < 30 ml/min/1.73 m^2^. Individuals with lower eGFR values were older, had a slight male predominance, had a higher waist-to-hip ratio, as well as higher rates of hypertension, diabetes mellitus and dyslipidemia. Moreover, participants with lower eGFR covered significantly shorter 6-min test distance (*p-value* < 0.001), had worse timed chair rise stand test time (*p-value*: 0.016) and lower maximal grip strength (*p-value* < 0.001). Regarding albuminuria, 570 participants presented microalbuminuria, 50 had 24-h albumin excretion over 300 mg/24 h and 2 presented nephrotic-range albuminuria (> 3000 mg/24 h).

**Table 1:** Participants’ clinicodemographic and physical function characteristics, overall and by categories of estimated glomerular filtration rate

Variable	Overall (*n* = 7396)	Estimated glomerular filtration rate			*P*
		≥ 90 ml/min/1.73 m^2^ (*n* = 2702)	60–90 ml/min/1.73 m^2^ (*n* = 4262)	< 60 ml/min/1.73 m^2^ (*n* = 432)	
Age (years)	61 [53–66]	56 [52–62]	63 [56–68]	69 [65–72]	** < 0.001**
Male sex	3,732 (50.5)	1,396 (51.7)	2,092 (49.1)	244 (56.5)	** < 0.001**
Caucasian ethnicity	7,299 (98.7)	2,656 (98.3)	4,219 (99.0)	424 (98.1)	0.628
Educational level					
* Low*	2,547 (34.4)	813 (30.1)	1,511 (35.5)	223 (51.6)	0.094
* Medium*	2,050 (27.7)	819 (30.3)	1,129 (26.9)	102 (23.6)	
* High*	2,799 (37.8)	1,070 (39.6)	1,622 (38.1)	107 (24.8)	
Alcohol consumption					
* None*	1,345 (18.2)	470 (17.4)	764 (17.9)	111 (25.7)	0.140
* Low*	4,318 (58.4)	1,551 (57.4)	2,561 (60.1)	251 (58.1)	
* High*	1,733 (23.4)	681 (25.2)	982 (23.0)	70 (16.2)	
Smoking status					0.132
* Former*	3,658 (49.5)	1,266 (46.9)	2,148 (50.4)	244 (56.5)	
* Current*	971 (13.1)	432 (16.0)	502 (11.8)	37 (8.6)	
Lifetime smoking (packyears)	0.9 [0–16]	1.5 [0–17.5]	0.6 [0–15]	1 [0–18]	0.067
BMI (kg/m^2^)	26.3 [23.9–29.3]	26 [23.5–29.1]	26.3 [24–29.2]	28.1 [25.4–31.3]	0.317
Waist circumference (cm)	94.4 [85.5–103.5]	93.5 [84.5–102.3]	94.4 [85.7–103.5]	101 [91.9–110.6]	** < 0.001**
Waist-to-hip ratio	0.9 [0.9–1]	0.9 [0.9–1]	0.9 [0.9–1]	1 [0.9–1]	** < 0.001**
Limited mobility	1,565 (21.2)	475 (17.6)	904 (21.2)	186 (43.1)	0.249
Energy intake (kcal/day)	2070.6 [1761.8–2503]	2124 [1746.7–2574.9]	2045.8 [1709.5–2470]	1,982.7 [,615.3–2400.9]	**0.014**
Office SBP (mmHg)	132 [121–145]	131 [120–143]	133 [122–145]	139 [125–152]	** < 0.001**
Office DBP (mmHg)	75 [69–82]	75 [69–82]	75 [69–82]	74 [67–81]	0.052
Hypertension	3,999 (54.1)	1,281 (47.4)	2,368 (55.6)	350 (81.0)	**0.013**
Diabetes mellitus status					
* Prediabetes*	1,099 (14.9)	374 (13.8)	953 (22.4)	78 (18.1)	** < 0.001**
* Type 2 diabetes*	1,774 (24.0)	635 (23.5)	647 (15.2)	186 (43.1)	
* Other diabetes*	47 (0.6)	29 (1.1)	15 (0.4)	3 (0.7)	
Cardiovascular disease history	1,227 (16.6)	345 (12.8)	746 (17.5)	136 (31.5)	**0.021**
Serum LDL-C (mmol/l)	3 [2.3–3.7]	3 [2.3–3.7]	3 [2.4–3.8]	2.6 [1.9–3.4]	0.073
Serum HDL-C (mmol/l)	1.5 [1.2–1.8]	1.5 [1.2–1.8]	1.5 [1.2–1.8]	1.3 [1.1–1.7]	** < 0.001**
Serum triglycerides (mmol/l)	1.2 [0.87–1.7]	1.15 [0.83–1.66]	1.2 [0.89–1.68]	1.42 [1.02–2.01]	0.117
Use of lipid-modifying drugs	2,386 (32.3)	771 (28.5)	1,369 (32.1)	246 (56.9)	** < 0.001**
Albuminuria (mg/24 h)	5.59 [3.49–10.56]	5.82 [3.63–10.25]	5.34 [3.41–10.05]	8.64 [3.67–31.76]	0.068
eGFR (ml/min/1.73 m^2^)	84.70 [73.95–94.42]	97.62 [93.38–102.84]	78.49 [71.66–84.31]	53.90 [48.70–57.52]	NA
**Physical function**					
6-min walk distance (m)	591 [540–644]	603 [551–654]	588 [538–641]	533 [484.8–592.5]	** < 0.001**
Gait speed (m/s)	1.64 [1.50–1.79]	1.68 [1.53–1.82]	1.63 [1.49–1.78]	1.48 [1.35–1.65]	** < 0.001**
Timed chair stand test time (s)	24 [21–28]	23 [20–27]	24 [21–28]	27 [23–31]	**0.016**
Maximal grip strength (kg)	32 [26–42]	34 [27–43]	32 [26–41]	30 [24–38]	** < 0.001**
Elbow flexion strength (Nm)	55.68 [41.52–73.13]	56.84 [41.92–75.05]	55.28 [41.77–71.05]	52.92 [36.29–66.5]	0.436
Elbow extension strength (Nm)	36.11 [25.74–48.34]	36.73 [26.15–49.37]	35.52 [25.43–47.45]	35.55 [24.31–46.59]	0.446
Knee flexion strength (Nm)	41.48 [31.18–54.35]	43.00 [31.85–56.07]	39.97 [30.86–53.28]	36.50 [28.05–50.43]	0.339
Knee extension strength (Nm)	127.73 [99.48–161.32]	129.76 [100.55–164.27]	126.88 [99.29–159.72]	118.46 [93.42–143.40]	0.398

Data presented as median [interquartile range] or number (column percentage). Bold text indicates statistical significance

*P-values* refer to the comparisons of parameters across kidney function subgroups, using the chi-square or the Kruskal–Wallis test

*BMI* body mass index, *SBP* systolic blood pressure, *DBP* diastolic blood pressure, *LDL-C* low-density lipoprotein cholesterol, *HDL-C* high-density lipoprotein cholesterol, *eGFR* estimated glomerular filtration rate, *NA* not applicable

### Physical performance and eGFR

**Fig. 2:**
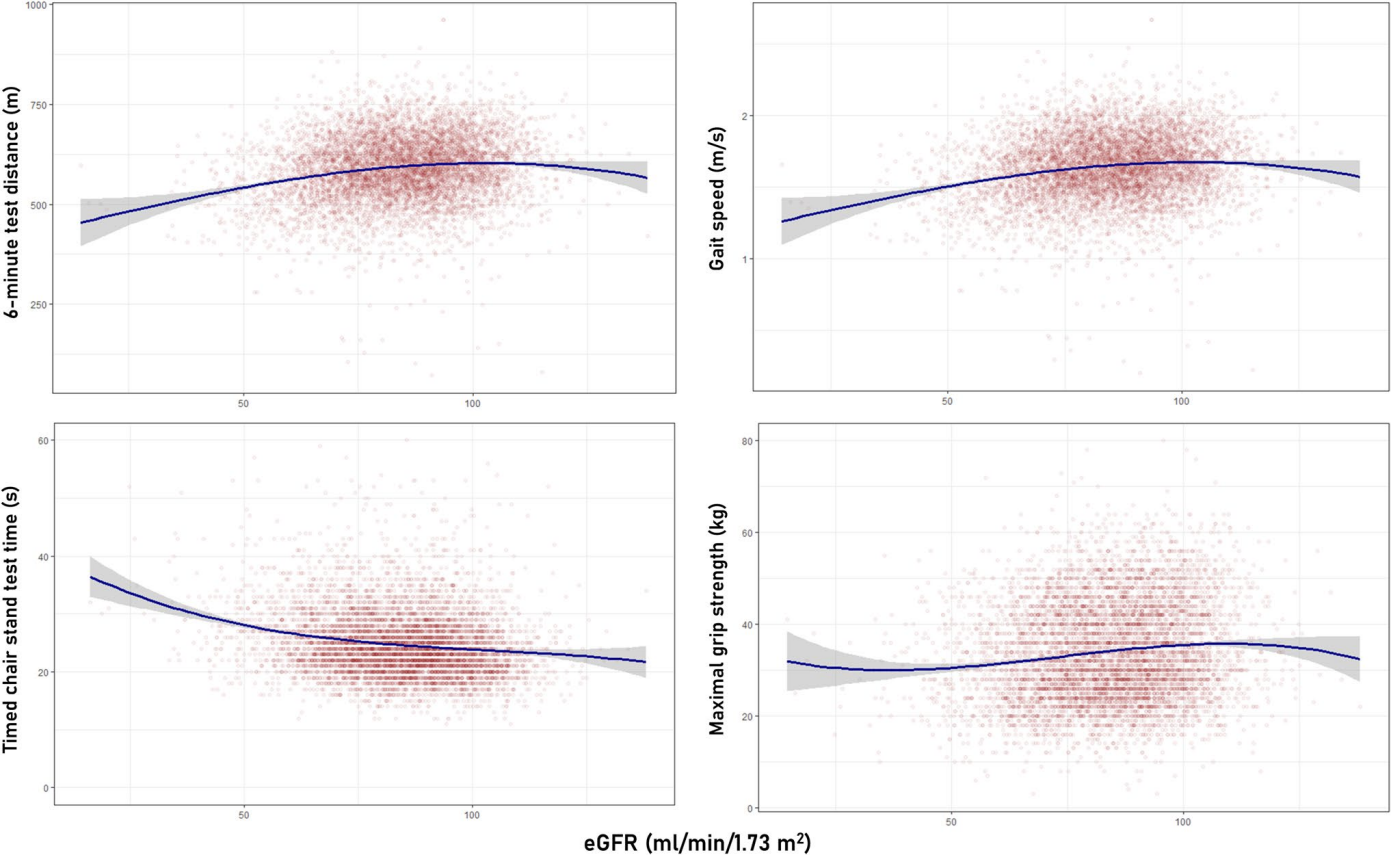
Association of estimated glomerular filtration rate with six-minute walk test distance, gait speed, timed chair rise stand test time and maximal grip strength

**Fig. 3:**
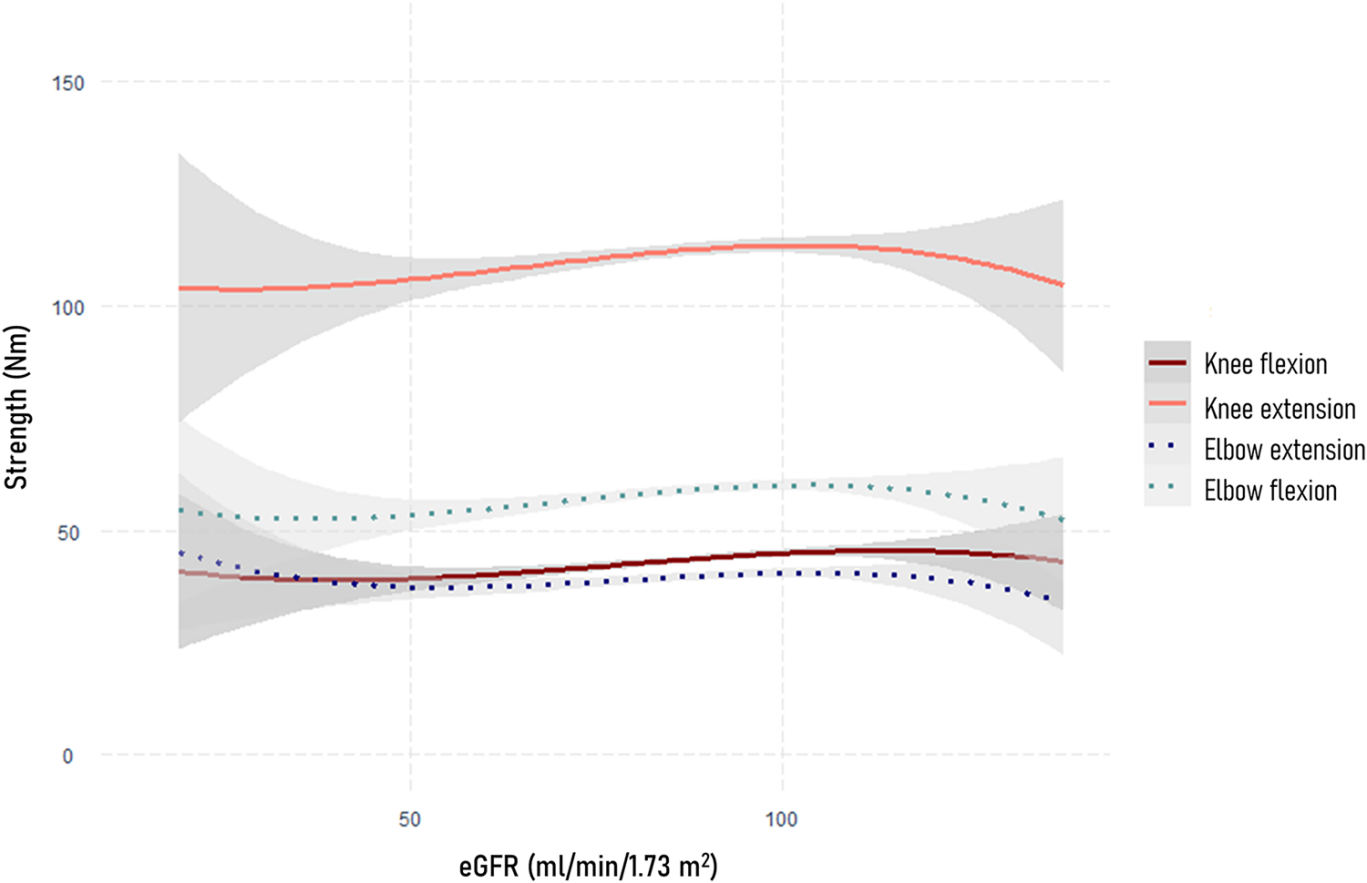
Association of estimated glomerular filtration rate with knee and elbow flexion and extension strength

The relationship between eGFR and 6-min test distance, gait speed, timed chair rise stand test time and maximal grip strength is depicted in Fig. 2. A non-linear relationship was observed between eGFR, 6-min test distance, gait speed and maximal grip strength, with both low and very high eGFR values being associated with worse physical performance. Multivariable regression analysis (Table 2) indicated that eGFR > 90 ml/min/1.73 m^2^ was associated with significantly shorter 6-min walk distance (*β*: -6.13 m, 95% confidence intervals-CI: -9.44 to -2.82, model 3) and slower gait speed (*β*: − 0.02 m/s, 95% CI − 0.03 to − 0.01, model 3), compared to eGFR 60–90 ml/min/1.73 m^2^. In addition, after adjusting for all covariates, eGFR < 60 ml/min/1.73 m^2^ was linked to significantly shorter 6-min walk distance (*β*: -13.04 m, 95% CI − 19.95 to − 6.13), slower gait speed (*β*: -0.04 m/s, 95% CI − 0.06 to − 0.02), worse timed chair rise stand test time (*β*: 0.91 s, 95% CI 0.36 to 1.47) and lower maximal grip strength (*β*: − 0.83 kg, 95% CI − 1.50 to − 0.15). Figure 3 illustrates the association of eGFR with isometric muscle strength of flexors and extensors of arms and legs, indicating that lower eGFR values correlate to worse isometric muscle strength. Multivariable regression analysis demonstrated that eGFR < 60 ml/min/1.73 m^2^ was linked to significantly lower elbow flexion strength (*β*: − 3.64 Nm, 95% CI − 7.11 to − 0.16), compared to eGFR 60–90 ml/min/1.73 m^2^.

**Table 2:** Multivariable linear regression analysis of the relationship of estimated glomerular filtration rate and albuminuria with physical performance markers

eGFR (vs. 60–90 ml/min/1.73 m^2^)	> 90 ml/min/1.73 m^2^			< 60 ml/min/1.73 m^2^		
	Model 1	Model 2	Model 3	Model 1	Model 2	Model 3
6-min test distance (m)	**− 3.73 (− 7.39; − 0.08)**	**− 6.13 (− 9.44; − 2.83)**	**− 6.13 (− 9.44; − 2.82)**	**− 18.67 (− 26.32; − 11.02)**	**− 13.04 (− 19.95; − 6.13)**	**− 13.04 (− 19.95; − 6.13)**
Gait speed (m/s)	**− 0.01 (− 0.02; − 0.00)**	**− 0.02 (− 0.03; − 0.01)**	**− 0.02 (− 0.03; − 0.01)**	**− 0.05 (− 0.07; − 0.03)**	**− 0.04 (− 0.06; − 0.02)**	**− 0.04 (− 0.06; − 0.02)**
Timed chair stand test time (s)	**− 0.31 (− 0.59; − 0.02)**	**− **0.17 (**− **0.45; 0.10)	− 0.17 (− 0.44; 0.11)	**1.38 (0.80; 1.95)**	**0.91 (0.35; 1.46)**	**0.91 (0.36; 1.47)**
Maximal grip strength (Kg)	− 0.05 (− 0.39; 0.29)	− 0.04 (− 0.38; 0.30)	− 0.05 (− 0.39; 0.28)	− **1.00 (**− **1.67; **− **0.32)**	− **0.83 (**− **1.50; **− **0.15)**	− **0.83 (**− **1.50; **− **0.15)**
Elbow flexion strength (Nm)	− 0.25 (− 1.71; 1.20)	− 0.12 (− 1.58; 1.34)	− 0.14 (− 1.60; 1.32)	− **4.27 (**− **7.75; **− **0.80)**	− **3.64 (**− **7.12; **− **0.17)**	− **3.64 (**− **7.11; **− **0.16)**
Elbow extension strength (Nm)	− 0.41 (− 1.78; 0.97)	− 0.27 (− 1.65; 1.11)	− 0.26 (− 1.64; 1.12)	− 0.42 (− 3.73; 2.88)	0.13 (− 3.18; 3.43)	0.12 (− 3.19; 3.44)
Knee flexion strength (Nm)	− 0.97 (− 2.10; 0.16)	− 0.64 (− 1.77; 0.49)	− 0.64 (− 1.77; 0.49)	− 2.47 (− 5.22; 0.28)	− 1.84 (− 4.57; 0.89)	− 1.84 (− 4.57; 0.89)
Knee extension strength (Nm)	− **3.67 (**− **6.40; **− **0.95)**	− 2.35 (− 5.02; 0.33)	− 2.39 (− 5.10; 0.28)	− **8.09 (**− **14.72; **− **1.46)**	− 6.14 (− 12.62; 0.34)	− 6.15 (− 12.63; 0.32)
*Albuminuria* *(vs. < 15 mg/24 h)*	*15–30 mg/24 h*			* > 30 mg/24 h*		
6-min test distance(m)	− **8.41 (**− **14.49; **− **2.33)**	− **5.91 (**− **11.41; **− **0.42)**	− **5.84 (**− **11.35; **− **0.33)**	− **21.56 (**− **27.93; **− **15.19)**	− **11.80 (**− **17.57; **− **6.03)**	− **12.48 (**− **18.28; **− **6.68)**
Gait speed (m/s)	− **0.02 (**− **0.04; **− **0.01)**	− **0.02 (**− **0.03; **− **0.00)**	− **0.02 (**− **0.03; **− **0.00)**	− **0.06 (**− **0.08; **− **0.04)**	− **0.03 (**− **0.05; **− **0.02)**	− **0.03 (**− **0.05; **− **0.02)**
Timed chair stand test time (s)	0.32 (− 0.14; 0.79)	0.20 (− 0.25; 0.65)	0.24 (− 0.21; 0.69)	**1.03 (0.55; 1.51)**	**0.60 (0.13; 1.07)**	**0.51 (0.11; 1.06)**
Maximal grip strength (Kg)	− 0.14 (− 0.70; 0.41)	− 0.20 (− 0.76; 0.35)	− 0.29 (− 0.84; 0.27)	− **1.20 (**− **1.78; **− **0.62)**	− **1.23 (**− **1.81; **− **0.66)**	− **1.34 (**− **1.91; **− **0.76)**
Elbow flexion strength (Nm)	− 1.55 (− 3.77; 0.67)	− 1.66 (− 3.88; 0.56)	− 1.76 (− 3.99; 0.46)	− **3.38 (**− **5.85; **− **0.92)**	− **3.19 (**− **5.66; **− **0.72)**	− **3.31 (**− **5.80; **− **0.82)**
Elbow extension strength (Nm)	− 1.41 (− 3.50; 0.69)	− 1.52 (− 3.61; 0.58)	− 1.49 (− 3.59; 0.60)	− 1.85 (− 4.18; 0.47)	− 1.73 (− 4.06; 0.60)	− 1.86 (− 4.21; 0.49)
Knee flexion strength (Nm)	0.96 (− 0.77; 2.70)	0.78 (− 0.94; 2.49)	0.77 (− 0.95; 2.49)	− 1.50 (− 3.40; 0.40)	− 1.58 (− 3.47; 0.31)	− 1.67 (− 3.57; 0.23)
Knee extension strength (Nm)	0.02 (− 4.16; 4.20)	− 0.79 (− 4.86; 3.28)	− 0.98 (− 5.06; 3.10)	− 1.87 (− 6.44; 2.69)	− 2.53 (− 7.00; 1.93)	− 3.09 (− 7.58; 1.40)

Data presented as *β* coefficient (95% confidence intervals). Bold text indicates statistical significance

Model 1 adjusts for age, sex, ethnicity, educational level, and diabetes mellitus

Model 2 adjusts *additionally* for body mass index, smoking, limited mobility and alcohol consumption

Model 3 adjusts *additionally* for systolic blood pressure. Albuminuria models also adjusted for eGFR

The association of renal hyperfiltration with physical function markers is exhibited in Suppl. Table 2. The presence of renal hyperfiltration was linked to significantly shorter 6-min walk distance (*β*: − 18.98 m, 95% CI − 33.09 to − 4.87) and slower gait speed (*β*: − 0.05 m/s, 95% CI − 0.09 to − 0.01). Subgroup analysis (Suppl. Table 3) indicated that eGFR_Cr_ > 90 ml/min/1.73 m^2^ was inversely associated with 6-min walk distance, gait speed, maximal grip strength, knee flexion and extension strength, while eGFR_Cr_ < 60 ml/min/1.73 m^2^ was linked to shorter 6-min walk distance, slower gait speed and worse timed chair rise stand test time. On the other hand, compared to eGFR_Cr-Cys_ 60–90 ml/1.73 m^2^, eGFR_Cr-Cys_ > 90 ml/min/1.73 m^2^ was associated with significantly lower maximal grip and knee extension strength, while eGFR_Cr-Cys_ < 60 ml/min/1.73 m^2^ was associated with significantly shorter 6-min walk distance, lower gait speed, elbow flexion and knee extension strength.

eGFR_Cr-Cys_ > 90 ml/min/1.73 m^2^ was inversely associated with maximal grip strength and knee extension strength, while eGFR_Cr-Cys_ < 60 ml/min/1.73 m^2^ was inversely associated with 6-min walk distance, gait speed, elbow flexion and knee extension strength.

### Physical performance and albuminuria

Multivariable regression analysis (Table 2) showed that urinary albumin excretion 15–30 mg/24 h was associated with shorter 6-min walk distance (*β*: − 5.84 m, 95% CI − 11.35 to − 0.33, model 3) and slower gait speed (*β*: − 0.02 m/s, 95% CI − 0.03 to − 0.00, model 3), compared to albuminuria < 15 mg/24 h. After adjusting for all covariates, urinary albumin excretion > 30 mg/24 h was linked to significantly shorter 6-min walk distance (*β*: − 12.48 m, 95% CI − 18.28 to − 6.68), slower gait speed (*β*: − 0.03 m/s, 95% CI − 0.05 to − 0.02), worse timed chair rise stand test time (*β*: 0.51 s, 95% CI 0.11–1.06), lower maximal grip (*β*: − 1.34 kg, 95% CI − 1.91 to − 0.76) and elbow flexion strength (*β*: − 3.31 Nm, 95% CI − 5.80 to − 0.82). No significant association was observed between albuminuria and elbow extension, knee flexion and extension strength.

### Sensitivity analysis

The outcomes of sensitivity analyses are presented in Table 3. Among participants without hypertension, eGFR > 90 ml/min/1.73 m^2^ was inversely associated with 6-min walk distance, gait speed and knee flexion strength, while urinary albumin excretion > 30 mg/24 h was linked to significantly lower knee flexion strength. Among individuals without dyslipidemia, eGFR > 90 ml/min/1.73 m^2^ was inversely associated with 6-min walk distance, gait speed and knee extension strength, while eGFR < 60 ml/min/1.73 m^2^ was linked to significantly lower knee flexion strength. Additionally, in this subpopulation, albuminuria > 30 mg/24 h was associated with significantly shorter 6-min walk distance, slower gait speed, lower maximal grip and elbow flexion strength.

**Table 3:** Sensitivity analysis of patients without hypertension or dyslipidemia

eGFR (vs. 60–90 ml/min/1.73 m^2^)	> 90 ml/min/1.73 m^2^		< 60 ml/min/1.73 m^2^	
	No hypertension	No dyslipidemia	No hypertension	No dyslipidemia
6-min test distance (m)	− **6.39 (**− **10.91; **− **1.86)**	− **6.38 (**− **10.19; **− **2.58)**	2.32 (− 11.82; 16.46)	− 3.27 (− 13.13; 6.58)
Gait speed (m/s)	− **0.02 (**− **0.03; **− **0.01)**	− **0.02 (**− **0.03; **− **0.01)**	0.01 (− 0.03; 0.06)	− 0.01 (− 0.04; 0.02)
Timed chair stand test time (s)	− 0.29 (− 0.65; 0.06)	− 0.27 (− 0.58; 0.04)	0.03 (− 1.06; 1.11)	− 0.27 (− 0.58; 0.04)
Maximal grip strength (Kg)	− 0.38 (− 0.85; 0.09)	− 0.27 (− 0.67; 0.13)	0.17 (− 1.27; 1.61)	− 0.17 (− 1.15; 0.82)
Elbow flexion strength (Nm)	− 0.99 (− 3.13; 1.15)	− 1.51 (− 3.32; 0.31)	− 9.50 (− 22.64; 3.63)	− 9.33 (− 15.95; − 2.71)
Elbow extension strength (Nm)	− − 0.90 (− 3.08; 1.29)	− 1.01 (− 2.76; 0.74)	1.27 (− 12.04; 14.58)	− 3.22 (− 9.67; 3.23)
Knee flexion strength (Nm)	− **1.98 (**− **3.63; **− **0.33)**	− 1.11 (− 2.50; 0.28)	− 0.09 (− 10.17; 9.87)	− **5.18 (**− **10.90; **− **0.54)**
Knee extension strength (Nm)	− 3.03 (− 7.05; 0.98)	− **4.13 (**− **7.42; **− **0.84)**	− 9.12 (− 33.68; 15.44)	− 11.68 (− 24.04; 0.67)
Albuminuria (vs. < 15 mg/24 h)	15–30 mg/24 h		> 30 mg/24 h	
6-min test distance (m)	− 7.91 (− 17.37; 1.56)	− **8.69 (**− **15.57; **− **1.81)**	− 0.54 (− 12.85; 11.76)	− **9.97 (**− **18.47; **− **1.47)**
Gait speed (m/s)	− 0.02 (− 0.05; 0.00)	− **0.02 (**− **0.04; **− **0.01)**	− 0.00 (− 0.04; 0.03)	− **0.03 (**− **0.05; **− **0.00)**
Timed chair stand test time (s)	0.26 (− 0.30; 0.82)	0.30 (− 0.44; 1.04)	− 0.89 (− 1.84; 0.05)	0.25 (− 0.42; 0.93)
Maximal grip strength (Kg)	0.04 (− 0.68; 0.75)	0.04 (− 0.68; 0.75)	− 0.55 (− 1.81; 0.71)	− **0.84 (**− **1.74; **− **0.01)**
Elbow flexion strength (Nm)	− 3.33 (− 7.34; 0.67)	− 1.61 (− 4.59; 1.37)	− 4.40 (− 10.25; 1.45)	− **4.72 (**− **8.71; **− **0.74)**
Elbow extension strength (Nm)	− 2.41 (− 6.48; 1.67)	− 1.31 (− 4.17; 1.55)	− 2.16 (− 8.08; 3.76)	− 3.49 (− 7.33; 0.34)
Knee flexion strength (Nm)	0.43 (− 2.63; 3.50)	− 0.85 (− 3.12; 1.43)	− **4.90 (**− **9.16; **− **0.64)**	− 1.29 (− 4.26; 1.68)
Knee extension strength (Nm)	− 3.40 (− 10.90; 4.10)	− 2.99 (− 8.39; 2.41)	− 1.95 (− 12.36; 8.46)	− 4.08 (− 11.11; 2.95)

Data presented as *β* coefficient (95% confidence intervals). Bold text indicates statistical significance

Models adjust for age, sex, ethnicity, educational level, diabetes mellitus, for body mass index, smoking, limited mobility, alcohol consumption, and systolic blood pressure. Albuminuria models also adjusted for eGFR

### Additional analyses

Stratified analysis based on sex (Suppl. Table 4) demonstrated that eGFR > 90 ml/min/1.73 m^2^ was associated with significantly lower elbow flexion, knee flexion and extension strength only among females (*P*_*interaction*_ < 0.05). Additionally, among males, eGFR > 90 ml/min/1.73 m^2^ was linked to significantly lower maximal grip strength (*P*_*interaction*_: 0.004), and urinary albumin excretion > 30 mg/24 was associated with significantly lower knee flexion strength (*P*_*interaction*_: 0.037).

Stratified analysis based on diabetes mellitus status (Suppl. Tables 5–6) indicated that among normoglycemic participants, eGFR > 90 ml/min/1.73 m^2^ was associated with significantly shorter 6-min walk distance and slower gait speed. Interestingly, among individuals with type 2 diabetes mellitus, eGFR > 90 ml/min/1.73 m^2^ was positively associated with elbow flexion and extension strength (*P*_*interaction*_ < 0.05). In diabetic participants, eGFR < 60 ml/min/1.73 m^2^ was linked to shorter 6-min walk distance, slower gait speed and worse timed chair rise stand test time. Albuminuria > 30 mg/24 h was associated with shorter 6-min walk distance and slower gait speed in participants with both prediabetes and type 2 diabetes mellitus. Among those with type 2 diabetes mellitus, urinary albumin excretion > 30 mg/24 h was also linked to worse timed chair rise stand test time and lower maximal grip strength.

## Discussion

The present cross-sectional analysis evaluated the link between physical performance and kidney function, aiming to delineate the relationship of various objective physical performance markers with glomerular filtration rate and albuminuria. The results of regression analyses suggested that both reduced eGFR (< 60 ml/min/1.73 m^2^) and increased albumin excretion (> 30 mg/24 h) were linked to poor physical performance and lower muscle strength of upper and lower extremities, as reflected by the worse outcomes in terms of gait speed, timed chair rise stand test times, maximal grip and elbow flexion strength. These associations were observed in both sexes, although it was more pronounced among males, especially regarding maximal grip strength. Similarly, among individuals with type 2 diabetes mellitus, reduced kidney function and albuminuria were associated with poor performance in the six-minute walk and timed chair rise stand tests.

The results of the present study confirm the findings of previous studies regarding the association between albuminuria and physical performance. Specifically, an analysis of the SPRINT cohort including normoglycemic individuals aged ≥ 75 years demonstrated that higher levels of albuminuria were linked to impaired self-reported functional status and slower gait speed [24]. Similarly, in the BRINK (Brain in Kidney Disease) cohort, urinary albumin excretion greater than 30 mg/24 h has been associated with impaired physical performance, assessed by the Short Physical Performance Battery score [25]. A higher degree of albuminuria was also linked to worse physical performance and lower grip strength in a cohort study of diabetic patients [26]. It should be noted that urinary albumin excretion has been associated with an increased risk of falls [27] and has been suggested to represent a predictor of sarcopenia [28]. Albuminuria has been also associated with impaired cognitive and executive functioning [29], while it has been proposed to reflect a proinflammatory state [30] predisposing to endothelial dysfunction [31]. Importantly, in the present study, low-grade albuminuria (15–30 mg/24 h) was associated with worse performance in the 6-min walk test, a finding that may reflect the importance of even subtle changes in urinary albumin excretion. This is in line with prior research proposing that mildly increased albuminuria is linked to higher risk of mortality [32], cardiovascular disease [33] and diabetes complications [34].

Prior research has demonstrated mixed results regarding the association of physical function with glomerular filtration rate. In particular, analyses of the SPRINT [24] and BRINK [25] cohorts have suggested that eGFR is not independently associated with physical performance status, after adjusting for demographics and comorbidities. On the other hand, in the Cardiovascular Health Study [35] including individuals aged ≥ 65 years, eGFR has emerged as a significant risk factor for physical function decline. Furthermore, patients with advanced pre-dialysis stages of chronic kidney disease have been shown to be at significant risk of poor physical performance and frailty, especially in the case of diabetes mellitus co-existence [36]. Worse physical function has been also suggested as a possible predictor of increased risk of hospitalization, major adverse cardiovascular events and progression to end-stage kidney disease [37]. Moreover, among patients with kidney failure, poor physical function has been linked to increased propensity to falls and worse quality of life, while sarcopenia may serve as a marker of higher premature mortality risk [38, 39].

Importantly, the present study demonstrated a non-linear relationship between eGFR and physical function, with eGFR values > 90 ml/min/1.73 m^2^ being linked to worse physical function, especially in the six-minute walk test. Serum creatinine originates from muscle tissue and hence low muscle mass may lead to lower endogenous creatinine production [40] and overestimation of eGFR_Cr_ [41]. In this context, serum creatinine has been shown to positively correlate to appendicular lean mass and grip strength, while the eGFR_Cys_-to-eGFR_Cr_ ratio has been suggested as a potential marker of malnutrition and sarcopenia among hospitalized patients [42]. However, in the present study, low muscle mass leading to spuriously elevated eGFR values could not fully explain the observed relationship since the obtained outcomes for eGFR_Cr_ and eGFR_Cr-Cys_ were broadly similar, with eGFR_Cr-Cys_ > 90 ml/min/1.73 m^2^ being also associated with significantly lower maximal grip and knee extension strength. Nevertheless, it should be noted that eGFR_Cr-Cys_ measurements were available in less than half of the participants, while they are not able to completely correct the overestimation of eGFR by low muscle mass.

Supranormal eGFR values, termed renal hyperfiltration, have been proposed to reflect vascular dysfunction and have been associated with an increased cardiovascular disease risk, comparable to that of patients with stage 3a chronic kidney disease [43]. A cross-sectional analysis of the KNHANES (Korea National Health and Nutrition Examination Survey) has suggested a potential link between renal hyperfiltration and reduced appendicular skeletal muscle, a marker indicative of sarcopenia [44]. The link between sarcopenia and renal hyperfiltration may be based on the interplay of various factors, including oxidative stress, insulin resistance and overactivation of the renin–angiotensin–aldosterone system [45, 46]. Interestingly, inflammation is not only associated with protein catabolism, but also with structural changes and increased permeability of the glomerulus that may potentiate the development of renal hyperfiltration [47]. In addition, high-intensity physical exercise has been shown to exert favorable effects in abnormally elevated glomerular filtration rate measured by plasma iohexol clearance, leading to lower rates of renal hyperfiltration [48]. Similarly, endurance and strength exercise have been shown to reverse hyperfiltration among patients with obesity. Interestingly, in the present analysis, participants with type 2 diabetes mellitus with high eGFR (> 90 ml/min/1.73 m^2^) presented significantly better upper extremity strength, while no statistically significant associations were observed among individuals with prediabetes. The explanation of this finding is less clear and may be associated with the distinct pathophysiology of glomerular hyperfiltration in the setting of diabetes mellitus. The prevalence of renal hyperfiltration is highest among diabetes patients, representing an early phenomenon in the course of the disease, attributed to a complex combination of hyperglycemia, kidney hypertrophy and renal hemodynamic alterations [49]. On the other hand, sarcopenia has been associated with longer duration of diabetes since long-term complications, especially diabetic polyneuropathy may potentiate sensory dysfunction and muscle weakness [50].

The present study has several strengths. This is a population-based study including a large sample of individuals with a wide spectrum of kidney function. Physical performance was quantified using a variety of objective tests and standardized measurements, allowing a comprehensive evaluation of upper and lower extremity muscle strength. Analyses took into account multiple clinical and demographic covariates, limiting possible confounding effects.

On the other hand, this study followed a cross-sectional design, precluding thus the drawing of safe causal inferences. This may be of particular importance, as previous research in the field of physical exercise and performance has treated kidney function both as the determinant and the outcome [51–53]. Glomerular filtration rate estimation with creatinine-based equations may be confounded in case of low muscle mass; therefore, apart from integrating serum cystatin-c in calculations, measuring glomerular filtration rate with more accurate methods, such as the clearance of exogenous markers, may be needed to confirm the observed associations. Recently, a race-free equation has been proposed as most appropriate for glomerular filtration rate estimation [54]; however, the present population consisted mostly of Caucasian individuals (98.8%) and thus the inclusion of race in eGFR calculation is not expected to serve as a remarkable source of bias. Furthermore, the excluded participants (*n* = 293) were slightly older with a greater percentage of hypertension and type 2 diabetes mellitus; thus, it should be acknowledged that the analysis was based on a healthier population, which may limit the general applicability of outcomes. Missing data were treated by listwise deletion in order to have available information for all parameters used in the multivariate models, leaving finally a large sample size since data regarding covariates were available for most participants (96.2%). However, the possibility of bias due to missing data could not be safely excluded, as the assumption of missing completely at random may not be completely satisfied. It should be also noted that the majority of participants presented normal kidney function or early-stage chronic kidney disease and hence generalizability of outcomes in patients with stage 4 or 5 chronic kidney disease and nephrotic-range albuminuria may be limited.

## Conclusions

This study supports a significant relationship between impaired kidney function and poor physical performance. This finding applied both for individuals with reduced glomerular filtration rate and high urinary albumin excretion and was observed in multiple tests of upper and lower extremity strength. A non-linear association between eGFR and physical function markers was proposed, with both low and high eGFR values being linked to worse performance, especially in the six-minute walk test. Further large-scale prospective studies are needed to confirm these outcomes, as well as to elucidate whether interventions focused on the amelioration of physical functioning may exert beneficial effects in kidney function preservation, cardiovascular protection and quality of life improvement among individuals with chronic kidney disease.

## Supplementary Information

Below is the link to the electronic supplementary material.

Supplementary file1 (DOCX 47 KB)

## Data Availability

Data are available from the corresponding author upon reasonable request.
