# Supplementary material for: Association of kidney function with physical performance: the Maastricht study
Source: J Nephrol. 2024 Apr 9;37(8):2293–306. doi: 10.1007/s40620-024-01933-8 (PMC11649829; doi:10.1007/s40620-024-01933-8)
Supplement: Supplementary file 1 — Supplementary file1 (DOCX 47 KB) [file 40620_2024_1933_MOESM1_ESM.docx]

**Supplementary Appendix**

**Contents**

[**Suppl. Table 1.** Clinicodemographic characteristics of included and excluded participants. 2](#_Toc147771272)

[**Suppl. Table 2.** Association of renal hyperfiltration with physical performance markers. 3](#_Toc147771273)

[**Suppl. Table 3**. Association of physical function markers with glomerular filtration rate, estimated using serum creatinine or the combination of serum creatinine and cystatin C. 4](#_Toc147771274)

[S**uppl. Table 4**. Analysis of the association between glomerular filtration rate, albuminuria and physical performance markers, stratified by sex. 5](#_Toc147771275)

[**Suppl. Table 5**. Analysis of the association between glomerular filtration rate, albuminuria and physical performance markers, stratified by diabetes mellitus status. 6](#_Toc147771276)

[**Suppl.** **Table** **6**. Statistical significance of interactions with diabetes mellitus status. 7](#_Toc147771277)

# Suppl. Table 1. Clinicodemographic characteristics of included and excluded participants.

| **Variable** | **Included**  **(n=7,396)** | ***Excluded*** | ***P*** |
| --- | --- | --- | --- |
|  |  | ***(n=293)*** |  |
| Age (years) | 61 [53-66] | 62 [55-68] | **0.017** |
| Male sex | 50.5 | 48.5 | 0.542 |
| Caucasian ethnicity | 98.7 | 98.6 | 1 |
| Educational level |  |  |  |
| *Low* | 34.4 | 46.9 | **0.002** |
| *Medium* | 27.7 | 23.5 |  |
| *High* | 37.8 | 29.6 |  |
| Alcohol consumption |  |  |  |
| *None* | 18.2 | 29.7 | **<0.001** |
| *Low* | 58.4 | 49.3 |  |
| *High* | 23.4 | 21.0 |  |
| Smoking status |  |  | **<0.001** |
| *Former* | 49.5 | 46.0 |  |
| *Current* | 13.1 | 24.3 |  |
| Lifetime smoking (packyears) | 0.9 [0-16] | 4.6 [0-24.3] | **0.008** |
| BMI (kg/m^2^) | 26.3 [23.9-29.3] | 27.6 [24.8-34.5] | **<0.001** |
| Waist circumference (cm) | 94.4 [85.5-103.5] | 99 [87.9-110.5] | **<0.001** |
| Waist-to-hip ratio | 0.9 [0.9-1] | 0.9 [0.9-1] | **0.008** |
| Limited mobility | 21.2 | 40.4 | **<0.001** |
| Energy intake (kcal/day) | 2070.6 [1761.8-2503] | 2153.2 [1684.2-2532.7] | 0.455 |
| Office SBP (mmHg) | 132 [121-145] | 136 [123-149] | **0.003** |
| Office DBP (mmHg) | 75 [69-82] | 74 [69-82] | 0.538 |
| Hypertension | 54.1 | 63.3 | **0.002** |
| Diabetes mellitus status |  |  |  |
| *Prediabetes* | 14.9 | 14.3 | **<0.001** |
| *Type 2 diabetes* | 24.0 | 40.6 |  |
| *Other diabetes* | 0.6 | 1.0 |  |
| Cardiovascular disease history | 16.6 | 32.4 | **<0.001** |
| Serum LDL-C (mmol/l) | 3 [2.3-3.7] | 2.8 [2.2-3.6] | 0.110 |
| Serum HDL-C (mmol/l) | 1.5 [1.2-1.8] | 1.4 [1.1-1.7] | **<0.001** |
| Serum triglycerides (mmol/l) | 1.2 [0.87-1.7] | 1.3 [0.9-1.8] | 0.076 |
| Use of lipid-modifying drugs | 32.3 | 40.5 | **0.004** |
| eGFR (ml/min/1.73 m^2^) | 84.7 [73.9-94.4] | 86.2 [72.8-96.6] | 0.581 |
| Albuminuria (mg/24h) | 5.59 [3.49-10.56] | 7.54 [4.35-13.71] | 0.076 |

Data presented as median [interquartile range] or column percentage.
Bold text indicates statistical significance.

*BMI: body mass index; SBP: systolic blood pressure; DBP: diastolic blood pressure; LDL-C: low-density lipoprotein cholesterol; HDL-C: high-density lipoprotein cholesterol; eGFR: estimated glomerular filtration rate*

# Suppl. Table 2. Association of renal hyperfiltration with physical performance markers.

| **Physical function marker** | **Renal hyperfiltration** | | |
| --- | --- | --- | --- |
|  | ***Model 1*** | ***Model 2*** | ***Model 3*** |
| 6-minute test distance (m) | -12.80 (-28.46; 2.85) | **-18.96 (-33.10; -4.89)** | **-18.98 (-33.09; -4.87)** |
| Gait speed (m/s) | -0.04 (-0.08; 0.01) | **-0.06 (-0.09; -0.01)** | **-0.05 (-0.09; -0.01)** |
| Timed chair stand test time (s) | -0.52 (-1.72; 0.69) | -0.18 (-1.34; 0.98) | -0.15 (-1.31; 1.01) |
| Maximal grip strength (Kg) | -0.09 (-1.52; 1.34) | 0.03 (-1.39; 1.45) | -0.07 (-1.49; 1.35) |
| Elbow flexion strength (Nm) | 1.01 (-3.47; 5.48) | 1.20 (-3.27; 5.66) | 1.11 (-3.36; 5.58) |
| Elbow extension strength (Nm) | -0.32 (-4.56; 3.91) | -0.15 (-4.38; 4.08) | -0.11 (-4.34; 4.12) |
| Knee flexion strength (Nm) | 0.34 (-3.16; 3.83) | 0.67 (-2.79; 4.12) | 0.66 (-2.81; 4.11) |
| Knee extension strength (Nm) | -4.66 (-12.99; 3.67) | -3.02 (-11.13; 5.09) | -3.27 (-11.35; 4.88) |

Renal hyperfiltration (age-adjusted eGFR >130 ml/min/1.73 m^2^) is compared to normal renal filtration (age-adjusted eGFR 60-90 ml/min/1.73 m^2^).
Data presented as *β* coefficient (95% confidence intervals). Bold text indicates statistical significance.
Model 1 adjusts for age, sex, ethnicity, educational level, and diabetes mellitus.
Model 2 adjusts *additionally* for body mass index, smoking, limited mobility and alcohol consumption.
Model 3 adjusts *additionally* for systolic blood pressure.

# Suppl. Table 3. Association of physical function markers with glomerular filtration rate, estimated using serum creatinine or the combination of serum creatinine and cystatin C.

| ***Creatinine  eGFR_CKD-EPI_*** | ***>90 ml/min/1.73 m^2^*** | | | ***<60 ml/min/1.73 m^2^*** | | |
| --- | --- | --- | --- | --- | --- | --- |
|  | ***Model 1*** | ***Model 2*** | ***Model 3*** | ***Model 1*** | ***Model 2*** | ***Model 3*** |
| 6-minute test distance (m) | **-10.04 (-13.77; -6.30)** | **-8.46 (-11.84; -5.08)** | **-8.46 (-11.84; -5.07)** | **-16.10 (-23.42; -8.78)** | **-11.43 (-18.05; -4.82)** | **-11.42 (-18.04; -4.82)** |
| Gait speed (m/s) | **-0.03 (-0.04; -0.02)** | **-0.02 (-0.03; -0.01)** | **-0.02 (-0.03; -0.01)** | **-0.04 (-0.07; -0.02)** | **-0.03 (-0.05; -0.01)** | **-0.03 (-0.05; -0.01)** |
| Timed chair stand test time (s) | 0.18 (-0.11; 0.47) | 0.14 (-0.14; 0.43) | 0.15 (-0.14; 0.43) | **1.04 (0.48; 1.59)** | **0.66 (0.12; 1.20)** | **0.67 (0.13; 1.21)** |
| Maximal grip strength (Kg) | **-0.87 (-1.21; -0.52)** | **-0.88 (-1.22; -0.53)** | **-0.88 (-1.23; -0.53)** | -0.37 (-1.03; 0.29) | -0.22 (-0.87; 0.44) | -0.23 (-0.88; 0.43) |
| Elbow flexion strength (Nm) | **-1.50 (-2.97; -0.04)** | -1.31 (-2.77; 0.16) | -1.31 (-2.78; 0.15) | -0.58 (-3.69; 2.53) | -0.25 (-3.36; 2.85) | -0.27 (-3.38; 2.83) |
| Elbow extension strength (Nm) | -1.03 (-2.41; 0.35) | -0.90 (-2.28; 0.49) | -0.89 (-2.27; 0.49) | 0.72 (-2.21; 3.65) | 1.01 (-1.92; 3.93) | 1.02 (-1.91; 3.95) |
| Knee flexion strength (Nm) | **-2.12 (-3.25; -0.98)** | **-1.97 (-3.10; -0.85)** | **-1.97 (-3.10; -0.85)** | -0.68 (-3.14; 1.78) | -0.44 (-2.88; 2.00) | -0.44 (-2.89; 1.99) |
| Knee extension strength (Nm) | **-5.65 (-8.38; -2.92)** | **-5.31 (-7.98; -2.64)** | **-5.33 (-8.00; -2.66)** | -1.95 (-7.88; 3.98) | -1.19 (-6.97; 4.58) | -1.27 (-7.05; 4.50) |
| ***Creatinine-Cystatin C eGFR_CKD-EPI_*** | ***>90 ml/min/1.73 m^2^*** | | | ***<60 ml/min/1.73 m^2^*** | | |
| 6-minute test distance (m) | 1.27 (-4.59; 7.12) | -3.91 (-9.26; 1.45) | -3.92 (-9.28; 1.44) | **-33.32 (-47.63; -19.00)** | **-23.81 (-36.88; -10.73)** | **-23.79 (-36.87; -10.72)** |
| Gait speed (m/s) | 0.00 (-0.01; 0.02) | -0.01 (-0.03; 0.00) | -0.01 (-0.03; 0.00) | **-0.09 (-0.13; -0.05)** | **-0.07 (-0.10; -0.03)** | **-0.07 (-0.10; -0.03)** |
| Timed chair stand test time (s) | -0.41 (-0.85; 0.02) | -0.25 (-0.67; 0.18) | -0.24 (-0.67; 0.18) | **1.23 (0.19; 2.26)** | 0.59 (-0.41; 1.60) | 0.59 (-0.42; 1.59) |
| Maximal grip strength (Kg) | **-0.56 (-1.07; -0.06)** | **-0.54 (-1.05; -0.04)** | **-0.56 (-1.06; -0.05)** | **-1.20 (-2.35; -0.05)** | -0.93 (-2.09; 0.22) | -0.92 (-2.07; 0.24) |
| Elbow flexion strength (Nm) | -0.67 (-2.17; 0.82) | -0.52 (-2.02; 0.98) | -0.55 (-2.05; 0.95) | **-4.81 (-8.29; -1.33)** | **-4.19 (-7.67; -0.70)** | **-4.17 (-7.65; -0.68)** |
| Elbow extension strength (Nm) | -0.35 (-1.76; 1.07) | -0.20 (-1.62; 1.22) | -0.19 (-1.61; 1.23) | -0.69 (-4.01; 2.62) | -0.12 (-3.44; 3.21) | -0.12 (-3.45; 3.20) |
| Knee flexion strength (Nm) | **-1.21 (-2.38; -0.04)** | -0.88 (-2.05; 0.28) | -0.89 (-2.05; 0.28) | -2.75 (-5.51; 0.01) | -2.12 (-4.87; 0.62) | -2.13 (-4.87; 0.62) |
| Knee extension strength (Nm) | **-4.27 (-7.10; -1.45)** | **-3.03 (-5.80; -0.25)** | **-3.08 (-5.86; -0.30)** | **-9.03 (-15.72; -2.33)** | **-7.20 (-13.75; -0.64)** | **-7.21 (-13.77; -0.66)** |

Data presented as *β* coefficient (95% confidence intervals). Bold text indicates statistical significance.
Participants with eGFR 60-90 ml/min/1.73 m^2^ served as the reference group.

Model 1 adjusts for age, sex, ethnicity, educational level, and diabetes mellitus.

Model 2 adjusts *additionally* for body mass index, smoking, limited mobility and alcohol consumption.

Model 3 adjusts *additionally* for systolic blood pressure

# Suppl. Table 4. Analysis of the association between glomerular filtration rate, albuminuria and physical performance markers, stratified by sex.

| **Physical function marker** | **Estimated glomerular filtration rate** | | **Albuminuria** | |
| --- | --- | --- | --- | --- |
|  | ***>90 ml/min/1.73 m^2^*** | ***<60 ml/min/1.73 m^2^*** | ***15-30 mg/24h*** | ***>30 mg/24h*** |
| **6-minute test distance (m)** |  |  |  |  |
| *Male* | **-5.55 (-10.07; -1.03)** | **-13.40 (-22.63; -4.19)** | **-7.89 (-14.97; -0.81)** | **-10.56 (-17.31; -3.80)** |
| *Female* | **-6.73 (-11.30; -2.16)** | **-12.53 (-22.77; -2.29)** | -2.54 (-11.22; 6.14) | **-18.35 (-29.35; -7.34)** |
| *P for interaction* | 0.710 | 0.900 | 0.347 | 0.232 |
| **Gait speed (m/s)** |  |  |  |  |
| *Male* | **-0.02 (-0.03; -0.00)** | **-0.04 (-0.06; -0.01)** | **-0.02 (-0.04; -0.00)** | **-0.03 (-0.05; -0.01)** |
| *Female* | **-0.02 (-0.03; -0.01)** | **-0.03 (-0.06; -0.01)** | -0.19 (-0.52; 0.90) | **-0.05 (-0.08; -0.02)** |
| *P for interaction* | 0.710 | 0.900 | 0.347 | 0.232 |
| **Timed chair stand test time (s)** | | | | |
| *Male* | 0.09 (-0.29; 0.46) | **0.80 (0.05; 1.54)** | 0.28 (-0.31; 0.86) | **0.61 (0.07; 1.16)** |
| *Female* | **-0.43 (-0.81; -0.05)** | **1.08 (0.26; 1.90)** | 0.16 (-0.55; 0.87) | 0.50 (-0.38; 1.39) |
| *P for interaction* | 0.051 | 0.612 | 0.854 | 0.833 |
| **Maximal grip strength (Kg)** |  |  |  |  |
| *Male* | 0.29 (-0.18; 0.76) | **-1.65 (-2.55; -0.76)** | -0.69 (-1.40; 0.01) | **-1.58 (-2.25; -0.91)** |
| *Female* | -0.40 (-0.87; 0.07) | 0.27 (-0.73; 1.28) | 0.31 (-0.56; 1.19) | -0.79 (-1.88; 0.31) |
| *P for interaction* | **0.034** | **0.004** | 0.078 | 0.220 |
| **Elbow flexion strength (Nm)** |  |  |  |  |
| *Male* | 1.79 (-0.17; 3.75) | -3.43 (-7.86; 0.99) | -1.65 (-4.50; 1.21) | **-4.57 (-7.49; -1.65)** |
| *Female* | **-2.19 (-4.20; -0.17)** | -3.53 (-9.01; 1.95) | -2.06 (-5.56; 1.44) | -0.06 (-4.61; 4.50) |
| *P for interaction* | **0.004** | 0.977 | 0.858 | 0.096 |
| **Elbow extension strength (Nm)** | | | | |
| *Male* | 0.55 (-1.29; 2.40) | -2.01 (-6.23; 2.21) | -1.78 (-4.47; 0.91) | **-3.09 (-5.83; -0.34)** |
| *Female* | -1.14 (-3.04; 0.77) | 3.62 (-1.59; 8.84) | -1.18 (-4.49; 2.13) | 1.28 (-3.06; 5.63) |
| *P for interaction* | 0.193 | 0.096 | 0.781 | 0.090 |
| **Knee flexion strength (Nm)** |  |  |  |  |
| *Male* | 0.51 (-1.00; 2.02) | -1.36 (-4.78; 2.07) | 0.40 (-1.80; 2.60) | **-2.90 (-5.12; -0.68)** |
| *Female* | **-1.89 (-3.44; -0.33)** | -2.33 (-6.75; 2.08) | 1.21 (-1.51; 3.93) | 1.44 (-2.06; 4.94) |
| *P for interaction* | **0.024** | 0.730 | 0.649 | **0.037** |
| **Knee extension strength** |  |  |  |  |
| *Male* | 0.26 (-3.32; 3.84) | **-8.70 (-16.85; -0.56)** | -1.97 (-7.20; 3.25) | -4.60 (-9.86; 0.67) |
| *Female* | **-5.21 (-8.88; -1.52)** | -1.16 (-11.55; 9.24) | 0.41 (-6.04; 6.86) | 0.48 (-7.75; 8.70) |
| *P for interaction* | **0.029** | 0.258 | 0.571 | 0.301 |

Data presented as *β* coefficient (95% confidence intervals). Bold text indicates statistical significance. Models adjust for age, ethnicity, educational level, diabetes mellitus, body mass index, smoking, limited mobility, alcohol consumption and systolic blood pressure. Albuminuria models also adjusted for eGFR.

# Suppl. Table 5. Analysis of the association between glomerular filtration rate, albuminuria and physical performance markers, stratified by diabetes mellitus status.

| **Physical function marker** | **Estimated glomerular filtration rate** | | **Albuminuria** | |
| --- | --- | --- | --- | --- |
|  | ***>90 ml/min/1.73 m^2^*** | ***<60 ml/min/1.73 m^2^*** | ***15-30 mg/24h*** | ***>30 mg/24h*** |
| **6-minute test distance (m)** |  |  |  |  |
| Normal glucose metabolism | **-8.90 (-12.96; -4.85)** | **-15.06 (-25.36; -4.75)** | -6.39 (-14.40; 1.63) | -7.89 (-17.24; 1.47) |
| Prediabetes | 3.03 (-5.31; 11.37) | 2.52 (-13.33; 18.38) | -7.45 (-21.17; 6.27) | **-26.68 (-42.61; -10.74)** |
| Type 2 diabetes mellitus | **-**4.02 (-10.81; 2.75) | **-17.08 (-28.50; -5.66)** | -3.83 (-12.96; 5.30) | **-12.12 (-20.51; -3.73)** |
| **Gait speed (m/s)** |  |  |  |  |
| Normal glucose metabolism | **-0.02 (-0.04; -0.01)** | **-0.04 (-0.07; -0.01)** | -0.02 (-0.04; 0.00) | -0.02 (-0.05; 0.00) |
| Prediabetes | -0.01 (-0.02; 0.03) | 0.01 (-0.04; 0.03) | -0.02 (-0.06; 0.02) | **-0.07 (-0.12; -0.03)** |
| Type 2 diabetes mellitus | -0.01 (-0.03; 0.01) | **-0.05 (-0.08; -0.02)** | -0.01 (-0.04; 0.02) | **-0.03 (-0.06; -0.01)** |
| **Timed chair stand test time (s)** | | | | |
| Normal glucose metabolism | -0.21 (-0.55; 0.13) | 0.76 (-0.10; 1.62) | 0.29 (-0.37; 0.95) | -0.09 (-0.86; 0.68) |
| Prediabetes | -0.40 (-1.09; 0.29) | 0.21 (-1.04; 1.46) | 0.08 (-1.05; 1.20) | 0.81 (-0.50; 2.12) |
| Type 2 diabetes mellitus | 0.11 (-0.44; 0.67) | **1.45 (0.55; 2.34)** | 0.20 (-0.54; 0.95) | **0.88 (0.21; 1.54)** |
| **Maximal grip strength (Kg)** |  |  |  |  |
| Normal glucose metabolism | -0.08 (-0.50; 0.35) | -0.59 (-1.63; 0.46) | 0.47 (-0.35; 1.28) | -0.56 (-1.52; 0.40) |
| Prediabetes | -0.71 (-1.57; 0.14) | -1.01 (-2.56; 0.55) | **-1.68 (-3.05; -0.31)** | -0.25 (-1.86; 1.36) |
| Type 2 diabetes mellitus | 0.42 (-0.26; 1.10) | -0.89 (-1.95; 0.17) | -0.56 (-1.45; 0.34) | **-2.02 (-2.83; -1.21)** |
| **Elbow flexion strength (Nm)** |  |  |  |  |
| Normal glucose metabolism | -1.76 (-3.62; 0.10) | -5.03 (-12.02; 1.96) | -2.17 (-5.53; 1.18) | -2.17 (-6.61; 2.28) |
| Prediabetes | -1.56 (-5.11; 1.99) | -2.83 (-11.81; 6.15) | -3.46 (-9.47; 2.56) | -5.13 (-12.14; 1.88) |
| Type 2 diabetes mellitus | **3.98 (1.29; 6.66)** | -1.08 (-5.67; 3.50) | -0.05 (-3.48; 3.38) | -2.70 (-6.07; 0.66) |
| **Elbow extension strength (Nm)** | | | | |
| Normal glucose metabolism | -1.68 (-3.44; 0.07) | 2.12 (-4.57; 8.83) | 0.27 (-2.89; 3.43) | -0.50 (-4.71; 3.71) |
| Prediabetes | -1.91 (-5.26; 1.44) | -1.56 (-10.01; 6.88) | -5.11 (-10.77; 0.54) | -3.18 (-9.78; 3.41) |
| Type 2 diabetes mellitus | **3.37 (0.83; 5.90)** | 1.43 (-2.94; 5.80) | -1.58 (-4.83; 1.66) | -1.78 (-4.96; 1.40) |
| **Knee flexion strength (Nm)** |  |  |  |  |
| Normal glucose metabolism | -1.41 (-2.85; 0.03) | -4.89 (-10.66; 0.88) | -0.23 (-2.84; 2.38) | 1.45 (-1.95; 4.85) |
| Prediabetes | -1.71 (-4.45; 1.02) | -5.03 (-11.90; 1.84) | -1.16 (-5.76; 3.44) | -4.94 (-10.13; 0.24) |
| Type 2 diabetes mellitus | 1.47 (-0.61; 3.55) | 1.10 (-2.48; 4.68) | 2.44 (-0.21; 5.09) | -2.29 (-4.88; 0.30) |
| **Knee extension strength (Nm)** | | | | |
| Normal glucose metabolism | -4.40 (-7.80; -0.99) | 4.37 (-9.35; 18.09) | -0.50 (-6.69; 5.69) | -0.60 (-8.58; 7.37) |
| Prediabetes | -2.03 (-8.48; 4.42) | -14.98 (-31.32; 1.35) | -6.65 (-17.59; 4.29) | -9.29 (-21.45; 2.87) |
| Type 2 diabetes mellitus | 1.18 (-3.73; 6.10) | -5.37 (-13.81; 3.08) | 1.79 (-4.48; 8.05) | -1.84 (-7.99; 4.30) |

Data presented as *β* coefficient (95% confidence intervals). Bold text indicates statistical significance.
Models adjust for age, sex, ethnicity, educational level, body mass index, smoking, limited mobility, alcohol consumption and systolic blood pressure. Albuminuria models also adjusted for eGFR.

# Suppl. Table 6. Statistical significance of interactions with diabetes mellitus status.

| **Physical function marker** | **Estimated glomerular filtration rate** | | **Albuminuria** | |
| --- | --- | --- | --- | --- |
|  | ***>90 ml/min/1.73 m^2^*** | ***<60 ml/min/1.73 m^2^*** | ***15-30 mg/24h*** | ***>30 mg/24h*** |
| **6-minute test distance (m)** |  |  |  |  |
| Prediabetes | **0.011** | 0.067 | 0.896 | **0.046** |
| Type 2 diabetes mellitus | 0.216 | 0.795 | 0.646 | 0.506 |
| **Gait speed (m/s)** |  |  |  |  |
| Prediabetes | **0.011** | 0.067 | 0.896 | **0.046** |
| Type 2 diabetes mellitus | 0.216 | 0.795 | 0.646 | 0.506 |
| **Timed chair stand test time (s)** | | | | |
| Prediabetes | 0.617 | 0.475 | 0.718 | 0.243 |
| Type 2 diabetes mellitus | 0.325 | 0.274 | 0.864 | 0.060 |
| **Maximal grip strength (Kg)** |  |  |  |  |
| Prediabetes | 0.180 | 0.659 | **0.008** | 0.744 |
| Type 2 diabetes mellitus | 0.216 | 0.690 | 0.097 | **0.022** |
| **Elbow flexion strength (Nm)** |  |  |  |  |
| Prediabetes | 0.919 | 0.704 | 0.715 | 0.482 |
| Type 2 diabetes mellitus | **<0.001** | 0.352 | 0.386 | 0.850 |
| **Elbow extension strength (Nm)** | | | | |
| Prediabetes | 0.904 | 0.501 | 0.103 | 0.500 |
| Type 2 diabetes mellitus | **0.001** | 0.863 | 0.422 | 0.633 |
| **Knee flexion strength (Nm)** |  |  |  |  |
| Prediabetes | 0.843 | 0.976 | 0.730 | **0.042** |
| Type 2 diabetes mellitus | **0.021** | 0.083 | 0.160 | 0.084 |
| **Knee extension strength (Nm)** | | | | |
| Prediabetes | 0.518 | 0.075 | 0.337 | 0.240 |
| Type 2 diabetes mellitus | 0.059 | 0.234 | 0.611 | 0.808 |

Data represent *p-values*.
Bold text indicates an association, significantly different from that observed in participants with normal glucose metabolism.
Models adjust for age, sex, ethnicity, educational level, body mass index, smoking, limited mobility, alcohol consumption and systolic blood pressure. Albuminuria models also adjusted for eGFR.
